# Seasonal Patterns in Incidence and Antimicrobial Resistance of Common Bacterial Pathogens in Nursing Home Patients and Their Rooms

**DOI:** 10.3389/fpubh.2021.671428

**Published:** 2021-07-12

**Authors:** Marco Cassone, Julia Mantey, Kyle J. Gontjes, Bonnie J. Lansing, Kristen E. Gibson, Joyce Wang, Lona Mody

**Affiliations:** ^1^Division of Geriatric and Palliative Care Medicine, Department of Internal Medicine, Michigan Medicine, Ann Arbor, MI, United States; ^2^School of Public Health, University of Michigan, Ann Arbor, MI, United States; ^3^Department of Microbiology and Immunology, Michigan Medicine, Ann Arbor, MI, United States; ^4^Geriatric Research and Education Clinical Center, VA Ann Arbor, Ann Arbor, MI, United States

**Keywords:** VRE, *S. aureus*, *E. coli*, antimicrobial resistance, nursing homes, seasonality

## Abstract

**Background:** Colonization is the main precursor to infection, which may lead to adverse clinical outcomes among older adults in nursing homes (NHs). Understanding seasonal changes in the local burden of common bacterial pathogens is key to implementing appropriate and cost-effective infection prevention measures in this resource-constrained healthcare environment. It is thus surprising that seasonal trends in patient and environmental colonization with major bacterial pathogens are presently unknown in the expanding NH setting.

**Methods:** We examined the seasonal incidence of four major pathogens among 640 nursing home patients and high-touch surfaces within their rooms over 2 years. In cases where a significant number of antimicrobial-resistant strains was found, incidence in antimicrobial-susceptible and antimicrobial-resistant isolates was compared, along with antibiotic use trends.

**Results:** We observed spring peaks in the incidence of vancomycin-resistant enterococci (1.70 peak to trough ratio for both patient and environmental isolates) and methicillin-resistant *Staphylococcus aureus* (1.95 peak to trough ratio for patient isolates, 1.50 for environmental isolates). We also observed summer peaks in *Klebsiella pneumoniae* (1.83 and 1.82 peak to trough ratio for patient and environmental isolates, respectively), and ciprofloxacin-resistant *Escherichia coli*. Susceptible *S. aureus* and *E. coli* did not follow seasonal patterns.

**Conclusions:** A meaningful seasonal pattern may be present in the NH setting for several significant pathogens, and especially antimicrobial-resistant ones. Whether such patterns are consistent across geographic areas and over longer periods of time should be a key focus of investigation, in order to better inform timing of surveillance and infection prevention efforts in this setting.

## Introduction

Knowledge of bacterial pathogens' seasonal incidence patterns can improve endemic surveillance and outbreak preparedness, and inform public health interventions (1). Seasonal patterns for bacterial pathogens have been studied mostly in acute care settings, with community-based data evaluated to a lesser extent (1–3). Very rarely has data been included from nursing homes (NHs) (4), which host an expanding and high-risk patient population with specific risk factors, interactions, and standards of care. Environmental contamination is a key indicator of patient colonization and adverse outcome risk in NHs (5, 6), and thus its seasonal patterns should be an object of investigation along with patient burden.

Using longitudinal microbial surveillance data of patients and their immediate environment, we aimed to investigate seasonal patterns in patient and environmental burden of several common bacterial pathogens in NHs. Furthermore, we compared the seasonal variations among antibiotic-resistant and susceptible isolates, and assessed associations with antibiotic use.

## Methods

Six hundred forty patients admitted between December 2013 and December 2015 in six southeast Michigan NHs were screened at multiple body sites (nares, oral cavity, hand, groin, perianal area, and indwelling device insertion if applicable), along with ten high-touch surfaces in their room (bed controls, nurse call button, side taple top and bottom surface, bed railing, divider curtain, TV remote control, toilet seat, wheelchair, walker) for vancomycin-resistant enterococci (VRE), methicillin-resistant and susceptible *Staphylococcus aureus* (MRSA and MSSA, respectively), *Klebsiella pneumoniae*, and *Escherichia coli*, using previously described clinical sampling and laboratory identification protocols (7). Patients were eligible if above age 21 and newly admitted to the nursing home (within the past 14 days). Non-english speaking patients and patients in end-of-life care were not eligible. Informed consent was obtained for all patients. Methicillin resistance in *S. aureus*, as well as ceftazidime, ciprofloxacin, and imipenem resistance in gram-negative isolates was tested using the Kirby-Bauer disc diffusion method according to CLSI criteria as previously described (7).

Monthly incidence of each pathogen was calculated based on new acquisitions per eligible enrolled patient (visited on that month and not colonized with that specific pathogen). Incidence data from the different study years were then merged to obtain a single data point for each month of the year (3), and logistic regression analysis with categorical predictors was used to identify statistically significant incidence variations compared to an arbitrary reference month (September, chosen based on its average temperature falling in the middle of the yearly range in Michigan). A graphical representation of seasonal incidence variability was obtained by calculating and plotting the three-month moving average of incidence rates. When a substantial proportion of resistant isolates was found (ciprofloxacin-resistant vs. –susceptible *E. coli*, as well as MRSA vs. MSSA), the resistant-to-susceptible ratio of incidence was obtained (total number of incident resistant isolates, divided by the total number of incident susceptible isolates), and linear regression analysis and Pearson correlation coefficient were calculated to establish whether its variation were significant. The resistant-to-susceptible ratio was plotted alongside pathogen incidence as well as monthly antibiotic usage in the study facilities, defined as the percentage of patients receiving one or more doses of any antibiotic, or the specific antibiotic of interest, in that given month. Seasons were defined using the accepted meteorological convention for the northern hemisphere adopted by the majority of countries in the world (for the US, defined by the National Oceanic and Atmospheric Administration), i.e., Spring: March 1st–May 31st. Summer: June 1st–August 31st. Fall: September 1st–Nov*e*mber 30th. Winter: December 1st–February 28th.

## Results

During the 2-year period, a total of 266 VRE, 313 *S. aureus*, 333 *E. coli*, and 140 *K. pneumoniae* unique isolates were obtained from 640 patients. Four hundred and thirteen VRE, 696 *S. aureus*, 110 *E. coli*, and 154 *K. pneumoniae* unique isolates were obtained from environmental samples in patient rooms. The average incidence of VRE was 30.45 isolates per 1,000 patient days among patients, and 47.37 among environmental samples. For *S. aureus*, the average incidence was 23.66 and 51.36, respectively. For *E. coli*, patient and environmental average incidences were 29.77 and 9.86, and for *K. pneumoniae* 13.93 and 15.43, respectively.

**Figure 1:**
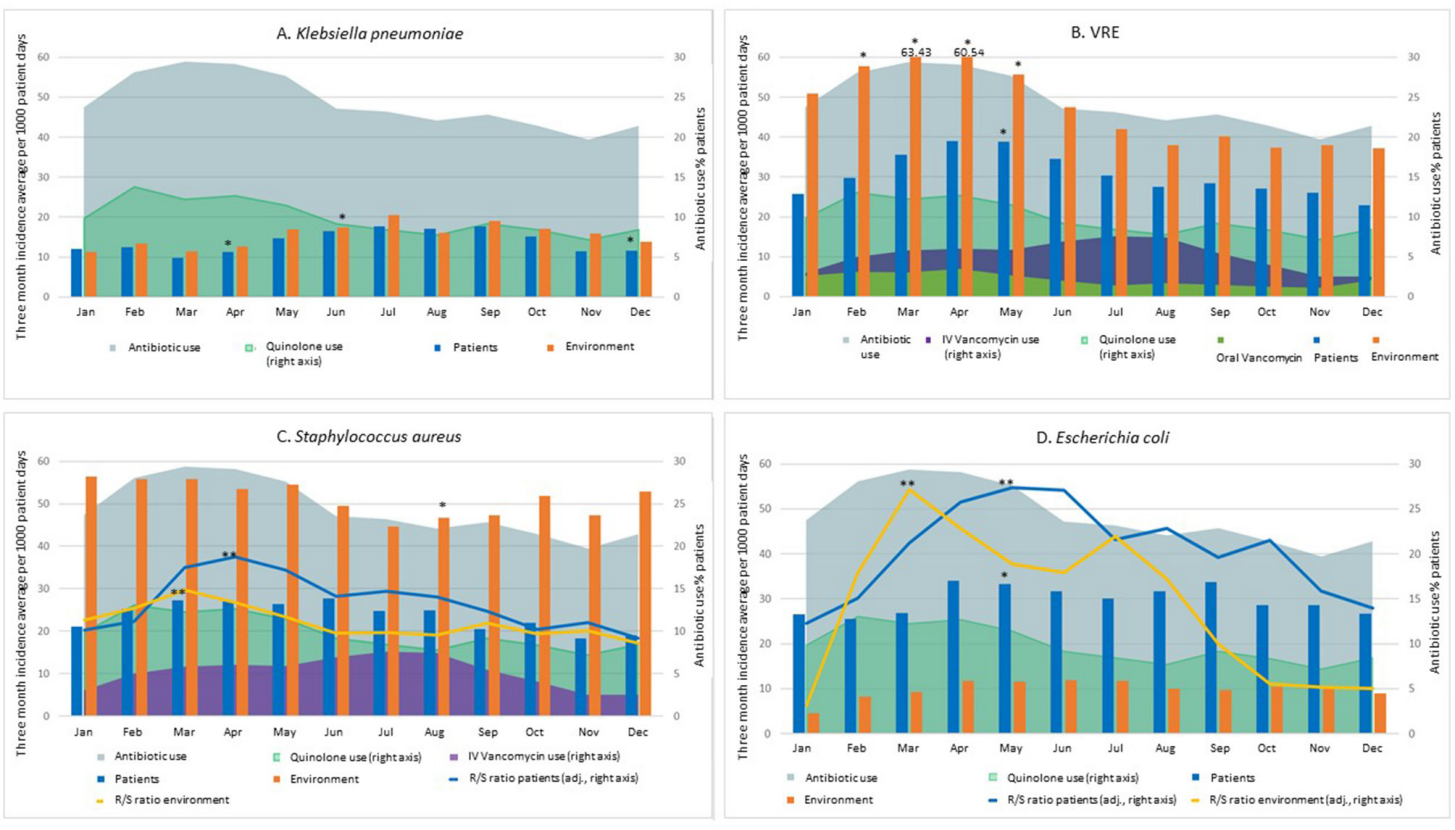
Seasonal incidence (3-month moving average) of patient and environmental isolates of *K. pneumoniae* **(A)**, VRE **(B)**, *S. aureus* **(C)**, and *E. coli* **(D)**. For *S. aureus*, the ratio of MRSA to MSSA isolates is also shown. For *E. coli*, the ciprofloxacin resistant to susceptible ratio is shown. Monthly facility usage of all antibiotic, ciprofloxacin and IV vancomycin (VRE and *S. aureus*) is also provided. Spring: March 1st–May 31st. Summer: June 1st–August 31st. Fall: September 1st–November 30th. Winter: December 1st–February 28th. The scale for the Resistant to Susceptible (R/S) ratio for *S. aureus* **(C)** is 1/10th of the right Y axis scale, for improved visibility. *p < 0.05 difference in single-month incidence compared to the arbitrary selected reference month (September) (logistic regression with categorical predictors). **p < 0.05 difference between peak and trough in the Resistant-to-Susceptible (R/S) ratio (linear regression and Pearson's correlation coefficient).

Seasonal variation was observed for *K. pneumoniae* among patients and the environment (Figure 1). For patient isolates, the 3-month average incidence reached a peak in summer (up to 17.74 new isolates per 1,000 patient days), while the trough was observed in early spring (9.71; *p* < 0.05; Figure 1A). For environmental isolates, both incidence rates and seasonal trends were similar to patient trends (peak incidence of 20.47 in summer, and trough of 11.27 in winter; *p* < 0.05), potentially because of frequent acquisitions and shedding between patients and their immediate environment. The majority of *K. pneumoniae* isolates were susceptible to all the tested antibiotics.

For VRE, patient and environmental incidence data followed similar seasonal patterns with a spring peak in both cases (Figure 1B). Specifically, 3-month incidence averages peaked at 63.43 per 1,000 patient days (environment) and 38.98 (patients), compared to 37.20 and 22.86 at the trough, respectively (70.5% increase from trough to peak in both cases) (*p* < 0.05 for both patient and environment).

In the case of *S. aureus* (Figure 1C), seasonal variations were not statistically significant for patient colonization: the three-month average incidence in patient colonization reached its highest in spring and early summer peaking at 27.67 new isolates per 1,000 patient days, versus a trough of 18.29 in late fall-early winter. Interestingly, higher *S. aureus* incidences were consistently reported for environmental isolates, for which a significant seasonal trend was observed (*p* < 0.05). This seasonal trend diverges when MRSA and MSSA are analyzed separately, as MRSA showed a seasonal trend (peak of 17.63 and 33.33 in late spring and trough of 9.05 and 22.15 in winter for patient and environmental isolates, respectively), while MSSA did not (hovering in a narrow range between a maximum of 11.89 and a minimum of 8.71 for patient isolates and between 28.49 and 22.44 for environmental isolates): the ratio of MRSA to MSSA shows a peak in spring and a trough in winter (ratio 0.96, *p* < 0.0001 and 0.81, *p* < 0.01 for patient isolates and for environmental isolates, respectively). Specifically, the peak ratio was 1.48 for environmental and 1.87 for patient isolates. In winter, instead, more MSSA were isolated than MRSA (ratio of 0.86 and 0.91, respectively).

Antimicrobial usage rates were higher in spring (peaking at 59% of all patients), and lowest in late fall—early winter (trough of 39%), and among specific classes quinolone usage followed a similar pattern (Figure 1). IV vancomycin usage rates increased following the increase in MRSA incidence. Specifically, they were at their lowest during winter (2.5% of all patients receiving IV vancomycin), doubled in the spring (5.75% of patients), and then increased an additional 30% in summer (7.6%) (Figure 1B). Oral vancomycin usage rates in the study facilities were at their highest in late winter and spring. Interestingly, this pattern was similar to the variations in VRE burden (Figure 1B).

For *E. coli*, we report a moderate seasonal variation for patient incidence (33% increase from trough to peak; *p* < 0.05). A similar trend appeared to be in place for environmental incidence, albeit with lower overall numbers. Among patient isolates, the 3-month average trough was 25.46 in winter, and the peak was 33.96 in spring (Figure 1D); among environmental isolates, the thorough and peak were 4.60 in winter, and 11.86 in summer. We observed an increase in the ratio of ciprofloxacin-resistant to ciprofloxacin–susceptible isolates (peak of 10.44 and 5.08 in summer and trough of 4.50 and 0.43 in winter for patient and environmental isolates, respectively). For patient incidence, the resistant to sensitive ratio was as high as 0.45 at peak, and as low as 0.20 during the winter through, while for environmental isolates the range was between 0.90 and 0.10 (ratio 0.95, *p* < 0.0001 for both patient and environmental isolates). Quinolone antibiotics use was lower in fall, with a minimum of 7% of all patients, and higher in spring (up to 13% of all patients, *p* < 0.01), with a tendency to precede the seasonal increase in *E. coli* incidence, and especially the increase in ciprofloxacin-resistant to -susceptible ratio (Figure 1D).

## Discussion and Conclusions

In the present study, we report seasonal variability in the incidence of several pathogens commonly found as colonizers and environmental contaminants in NHs, and compare patterns of patient and environmental burden variation. We also observed differences in seasonal patterns between pathogens susceptible and resistant to specific antimicrobials, and note their possible association with antimicrobial usage. Knowledge of seasonal patterns specific to post-acute care is key in outbreak surveillance, and in the planning and evaluation of new infection prevention measures and interventional studies.

Seasonality of bacterial infections has been investigated almost exclusively in acute care settings, and mostly limited to patient isolates, where seasonal trends have been established for several pathogens in a range of geographical settings (1, 2, 8, 9), including antimicrobial-resistant strains (10), often linking higher temperatures to increases in rates of infections, especially those due to gram-negative bacteria. Notably, a study focused on *S. aureus* infections reports seasonality in hospitals, but not in NHs (4), highlighting the importance of obtaining specific data in post-acute and long-term care.

Seasonality of colonization, on the other hand, has been rarely studied, despite its importance as both a vehicle of transmission and a precursor to infection. In a recent study conducted among intensive care units in 20 different US locations, seasonality in MRSA nasal colonization and VRE perianal colonization appeared to be driven by higher relative humidity values, while a higher latitude was positively associated with VRE colonization and negatively associated with MRSA colonization (11). Our present data show that the geographic area of our study (an ~50 km-radius area in southeast Michigan including the Detroit metropolitan area, its suburbs, and semi-rural locations at its outskirts) broadly fits into this pattern. A seasonal increase in MRSA colonization has recently been observed in a NH in Europe (12), as well as in healthy young populations (13). Reports from settings other than NHs are still very much relevant to NHs due to increased risk of introduction, especially in view of the current trends of diminishing length of stay and increase of resident turnover and new admissions.

We observed lower environmental contamination incidence with *E.coli* compared to patient colonization, while *K. pneumoniae* rates were generally similar between patients and their environment. For both *S. aureus* and VRE, instead, environmental contamination incidence was higher than patient colonization. Due to the scarcity of studies reporting both patient and environmental burden of common bacterial pathogens in the NH setting, we don't currently know if such trends are to be generally expected. Nevertheless, it is worth noting that residents in NHs tend to be more active and mobile than hospital patients, often undergo several social and/or therapeutic activities and sessions, and have more chances to interact with many potential environmental fomites.

Antibiotic use is an important consideration to bring into the picture, including the specific classes used. For example, we observed that the increase in MRSA incidence was paralleled by increased overall antibiotic use. In the literature, use of antibiotics lacking anti-MRSA activity has been shown to lead to an increase in nasal colonization with MRSA (14). In our case, we were able to demonstrate that this is accompanied by a significant relative decrease in MSSA, and also that this was reflected in our measures of environmental burden, too. Similarly, we demonstrated independent and distinct seasonal patterns for ciprofloxacin-susceptible and ciprofloxacin–resistant *E. coli*. Conversely, we did not observe significant antibiotic resistance in *K. pneumoniae* isolates, whose incidence did not increase parallel to antimicrobial usage, but rather with warmer months. This trend that has been reported before for this organism (1, 8), although this is the first time it has been found in the NH setting. Additionally, it is important to caution that although an association between resistance rates and antimicrobial use has been reported in some common gram-negative pathogens in various healthcare settings (15, 16), such association may present with a lag, especially when infections are used as the endpoint rather than colonization, presumably at least in part due to the time elapsed between colonization, infection, and eventually collection of clinical samples (15).

While we are still removed from a working knowledge of seasonality patterns in NHs at a nationally generalizable level, our findings constitute a step toward filling the present information void. Facility leadership and clinical decision makers should be aware of the higher risk of colonization with specific pathogens at specific times of the year, and consider active surveillance screening of patients at high risk of colonization, and/or high risk of adverse events from infection with such pathogens. Additionally, they should establish a rigorous, evidence-based antimicrobial stewardship program. Since pathogen seasonal trends have a local geographic component, clinical decision makers should either familiarize themselves with local data if available, or consider collecting and sharing new relevant information in their own facility.

Our study has strengths and limitations. Among the strengths, our observation that antimicrobial resistant strains may have different seasonal patterns compared to susceptible strains within the same species in the NH setting is an important springboard for further investigation. Such patterns carry important clinical practice implications, since a high proportion of such patients report current and/or recent antibiotic usage (17). Also, we tested multiple patient body sites, which has been demonstrated to greatly increase sensitivity (18). Among the limitations is the time span of the dataset, which encompasses only 2 years of observations, and thus prevented us from performing a time series analysis. Additionally, our study only assessed a limited geographic area. These limitations can only be overcome via further investigation with the ultimate goal of implementing tailored and measurable infection prevention policies in the expanding NH setting, which house a growing majority of at-risk patients.

## Data Availability Statement

The raw data supporting the conclusions of this article will be made available by the authors, without undue reservation.

## Ethics Statement

The studies involving human participants were reviewed and approved by University of Michigan Institutional Review Board. The patients/participants provided their written informed consent to participate in this study.

## Author Contributions

MC and LM conceived and planned the study. MC, BL, KEG, and KJG collected and entered study data and performed laboratory analysis of samples. MC, JM, and JW performed analysis and visualization of epidemiological data. All authors participated in the writing and critical review of the manuscript.

## Conflict of Interest

The authors declare that the research was conducted in the absence of any commercial or financial relationships that could be construed as a potential conflict of interest.
